# Abnormalities in the Fiber Composition and Capillary Architecture in the Soleus Muscle of Type 2 Diabetic Goto-Kakizaki Rats

**DOI:** 10.1100/2012/680189

**Published:** 2012-11-07

**Authors:** Shinichiro Murakami, Naoto Fujita, Hiroyo Kondo, Isao Takeda, Ryusuke Momota, Aiji Ohtsuka, Hidemi Fujino

**Affiliations:** ^1^Department of Physical Therapy, Himeji Dokkyo University, Himeji, Hyogo 670-8524, Japan; ^2^Department of Rehabilitation Science, Kobe University Graduate School of Health Sciences, Kobe 654-0142, Japan; ^3^Department of Human Morphology, Okayama University Graduate School of Medicine, Dentistry and Pharmaceutical Sciences, Okayama 700-8558, Japan; ^4^Department of Food Science and Nutrition, Nagoya Women's University, Nagoya-Shi, Aichi 467-8610, Japan; ^5^Department of Physical Therapy, Takarazuka University of Medical and Health Care, Takarazuka, Hyogo 666-0162, Japan

## Abstract

Type 2 diabetes mellitus is linked to impaired skeletal muscle glucose uptake and storage. This study aimed to investigate the fiber type distributions and the three-dimensional (3D) architecture of the capillary network in the skeletal muscles of type 2 diabetic rats. Muscle fiber type transformation, succinate dehydrogenase (SDH) activity, capillary density, and 3D architecture of the capillary network in the soleus muscle were determined in 36-week-old Goto-Kakizaki (GK) rats as an animal model of nonobese type 2 diabetes and age-matched Wistar (Cont) rats. Although the soleus muscle of Cont rats comprised both type I and type IIA fibers, the soleus muscle of GK rats had only type I fibers. In addition, total SDH activity in the soleus muscle of GK rats was significantly lower than that in Cont rats because GK rats had no high-SDH activity type IIA fiber in the soleus muscle. Furthermore, the capillary diameter, capillary tortuosity, and microvessel volume in GK rats were significantly lower than those in Cont rats. These results indicate that non-obese diabetic GK rats have muscle fiber type transformation, low SDH activity, and reduced skeletal muscle capillary content, which may be related to the impaired glucose metabolism characteristic of type 2 diabetes.

## 1. Introduction

Diabetic microangiopathy is one of the most common complications of diabetes and can manifest itself in multiple areas throughout the body. Damage to the microvessels in the kidney can lead to end-stage renal disease; structural alterations of the small vessels supplying nutrients and oxygen to peripheral nerves contribute to neuropathy; and damage to the microvasculature of the eye is the leading cause of diabetes-related vision loss [1]. In addition, microangiopathy also affects the skeletal muscle in diabetic patients, commonly presenting as a reduction in two-dimension capillary density [2]. 

Skeletal muscles are composed of heterogeneous types of fibers on the basis of the expression of different myosin heavy chain (MyHC) isoforms [3–7]. Skeletal muscle fibers that are categorized as slow-twitch oxidative (type I) contain only the slow MyHC isoform, whereas fast-twitch oxidative-glycolytic (type IIA) and fast-twitch glycolytic (type IIB) skeletal muscle fibers contain the fast MyHC isoforms. The contractile and metabolic properties of the skeletal muscle depend on its fiber type composition [3]. Fibers in the soleus muscle can be classified as either type I or type IIA fibers. 

Succinate dehydrogenase (SDH) is a key enzyme in the Krebs cycle, which occurs in muscle fibers. In general, the SDH activity of type I fibers is higher than that in type IIA and IIB fibers in skeletal muscle, whereas the SDH activity is higher in type IIA fibers than in type I fibers in the soleus muscle [8]. Therefore, there is an inverse correlation between fiber cross-sectional area and SDH activity for the different fiber types among the various muscles [9]. Decreased SDH activity has been reported in the skeletal muscles of patients and animal models of type 2 diabetes [7, 10, 11].

Diabetic human and animal subjects have been reported to show reduction in the content of type I fibers and an increase in the content of type IIA and IIB fibers in several muscles such as the vastus lateralis and the plantaris [7, 12–14]. In the soleus muscle of diabetic individuals with normal individuals, a lower percentage of type IIA fibers and lower oxidative enzyme activity in these fibers have been reported [7, 8, 10, 11]. These reports indicate that a shift occurs in low-oxidative-capacity type of muscle fibers and that muscle fiber type may affect the oxidative capacity in diabetic skeletal muscles. In addition, muscles with a high percentage of oxidative fibers have a greater capillary supply than low percentage of oxidative fibers [15–17]. Oxidative metabolism in the skeletal muscles may be an important factor relating to whole-body insulin sensitivity [13, 18] and the glucose level [7, 8, 10]. Muscle fibers in the skeletal muscles of patients with type 2 diabetes are reported to have reduced convective O_2_ delivery and diffusive O_2_ transport properties within muscle capillaries [19], leading to decreased capillary volume, lower levels of proangiogenic factors, and higher levels of antiangiogenic factors in the soleus muscle [20].

However, it is uncertain whether type 2 diabetes is linked to the altered muscle fiber types, SDH activity, and the capillary network in the skeletal muscles in diabetic individuals. In the present study, therefore, we examined the relationship between the fiber type distribution and the three-dimensional (3D) architecture of the capillary network in the soleus muscle of type 2 diabetic rats.

## 2. Materials and Methods

### 2.1. Animals

 All experiments were conducted in accordance with the National Institutes of Health (NIH publication no. 85-23, Revised 1,985) Guide for the Care and Use of Laboratory Animals (National Research Council, 1996) and approved by the Animal Care and Use Committee of Himeji Dokkyo University. Male GK rats aged 36 weeks (GK, *n* = 8) and age-matched male Wistar rats (Cont, *n* = 8) were used in this study. These rats were housed for 29 weeks in a room maintained under a controlled 12 h light-dark cycle at a temperature of 22 ± 2°C with 40–60% humidity. All rats were individually housed in same-sized cages, and food and water were provided ad libitum.

### 2.2. Muscle Preparation

 The muscle preparation procedure was described previously [17, 21, 22]. Briefly, animals were anesthetized with intraperitoneal administration of pentobarbital sodium (50 mg/kg). The left soleus muscles were excised, cleaned of excess fat and connective tissue, wet-weighed, frozen in isopentane precooled in liquid nitrogen, and stored at −80°C until further use. After the dissection of the left muscle, the abdominal cavity was opened, a blood sample was taken from the superior vena cava, the left common iliac artery and vein were ligated, and a catheter was inserted into the abdominal aorta to perfuse the right hindlimb with contrast medium. The right soleus muscles were first perfused for 3 min with 0.9% physiological saline containing 10,000 IU/L heparin at 37°C, followed by 10% glucose solution, and then the contrast medium, consisting of 2% fluorescent material (PUSR80; Mitsubishi Pencil, Tokyo, Japan), 8% gelatin (Nakalai Tesque, Kyoto, Japan), and distilled water. Following perfusion with contrast medium, the whole body of the rat was quickly immersed into cold saline for 10 min. Finally, the right soleus muscles were excised and frozen in isopentane precooled in liquid nitrogen.

### 2.3. Histochemical Procedures

 The midbelly of the left soleus muscle was mounted on a specimen chuck in Tissue Tek OCT compound. Serial transverse sections (10 *μ*m in thickness) were cut with a cryostat microtome (CM3050S; Leica Microsystems, Mannheim, Germany) at −20°C, and then, thawed to room temperature and air-dried for 30 min. To visualize the capillaries in the skeletal muscle, some sections were stained with alkaline phosphatase (AP) by incubation in 0.1%  *α*-naphthyl phosphate, 0.1% fast blue RR, and 0.01 M magnesium sulfate in 0.2 M borate buffer for 60 min at 37°C, and fixation with 10% formalin. Sections were observed with a light microscope (BX51; Olympus, Tokyo, Japan) and imaged with a CCD camera (VB-7000; Keyence, Osaka, Japan). The mean fiber cross-sectional area (FCSA,  *μ*m^2^) and the capillary density from AP staining were calculated using the NIH image software.

Some sections were also stained to determine the level of SDH activity in mitochondria, which is an indicator of mitochondrial oxidative capacity [9, 14]. For SDH histochemical analysis, sections were incubated in 0.1% nitroblue tetrazolium and 0.1 M sodium succinate in 0.1 M phosphate buffer (pH 7.2–7.6) for 30 min at 37°C and were dehydrated using ethanol. To determine SDH activity, we analyzed 100–200 fibers per muscle. The sectional images were visualized with a light microscope and imaged with a CCD camera. Each pixel was assigned a gray level value between 0 and 255, equivalent to 100% and 0% light transmission, respectively. The mean optical density of all pixels within a fiber was determined using a calibration photographic tablet with 21 steps of gradient density ranges and the corresponding diffused density values [17].

### 2.4. Immunohistochemistry

 Some sections were subjected to immunohistochemical staining protocols for fiber type classification by using the myosin skeletal slow antibody (NOQ7.5.4D; GeneTex, CA, USA; diluted 1 : 4000) incubated overnight at 4°C, followed by incubation with the fluorescein-conjugated AffiniPure donkey anti-mouse (H + L) secondary antibody (Jackson ImmunoResearch Laboratories, PA, USA) for 1 h at room temperature. Antibody binding was visualized with a fluorescent microscope (BX51) and imaged with a CCD camera.

### 2.5. ****3D Capillary Visualization

 The 3D capillary architecture was visualized using the fluorescent mode of a confocal laser scanning microscope (CLSM) (TCS-SP5, Leica Instruments) with an argon laser (488 nm) [17, 21, 22]. In brief, the sample block was sliced into 100 *μ*m sections using a cryostat. Images were obtained using the 20x objective lens, and each 100 *μ*m section was scanned to a depth of 50 *μ*m at 1 *μ*m per slice. Microscopic observations were performed in longitudinal sections. The CLSM images were automatically rendered and displayed as 3D images with a depth of 100 *μ*m. Digital images were converted into stack files for morphometric analysis to a depth of 100 *μ*m (NIH Image 1.63; NIH, Bethesda, MD, USA). The capillary volume of the skeletal muscle was measured in a square with 100 *μ*m sides and 50 *μ*m depth by using macros included in the NIH Image software [17, 21, 22]. Microvessel volume, number, and diameter were determined by measuring a 200 × 200-*μ*m^2^ area of 50 sections using NIH Image software.

### 2.6. Statistical Analysis

 All data were presented as the means ± SEM. All statistical tests were done using an unpaired Student's *t*-test. *P* < 0.05 indicates a significant difference.

## 3. Results

### 3.1. Characterization of GK Rats

**Figure 1:**
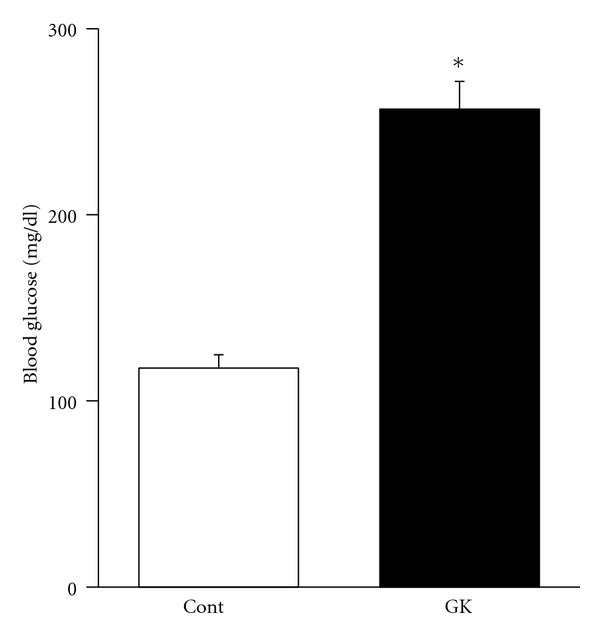
The mean blood glucose level in the Cont was 117.6 ± 7.1 mg/dL and that in the GK was 256.7 ± 15.0 mg/dL. The blood glucose level was significantly higher in the GK rats than in the Cont rats. *P < 0.05.

 Diabetic GK rats had significantly higher blood glucose levels compared to the Cont rats (Figure 1). Both the mean body mass and the mean soleus muscle mass were higher in the GK than the Cont rats, while the FCSA did not differ in the GK and Cont rats (Table 1). 

### 3.2. Distribution of Muscle Fiber Type

**Figure 2:**
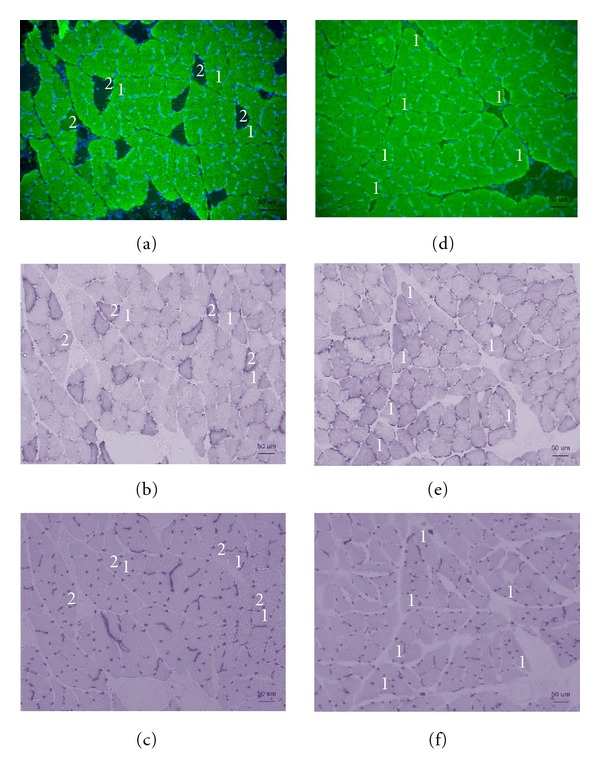
Serial transverse sections of the soleus muscle of the Cont (a–c) and GK (d–f) rats, stained for the myosin skeletal slow antibody (a), (d), SDH activities (b), (e), and AP activities (c), (f). 1, Type I fiber; 2, type II fiber. Scale bar: 50 *μ*m.

 The fiber-type distribution was confirmed by myosin heavy chain expression (Figures 2(a) and 2(d)). The soleus muscle of the Cont rats contained 83% type I fibers and 17% type II fibers, while the soleus muscle of the GK rats contained only type I fibers.

### 3.3. Oxidative Enzyme Activity

**Figure 3:**
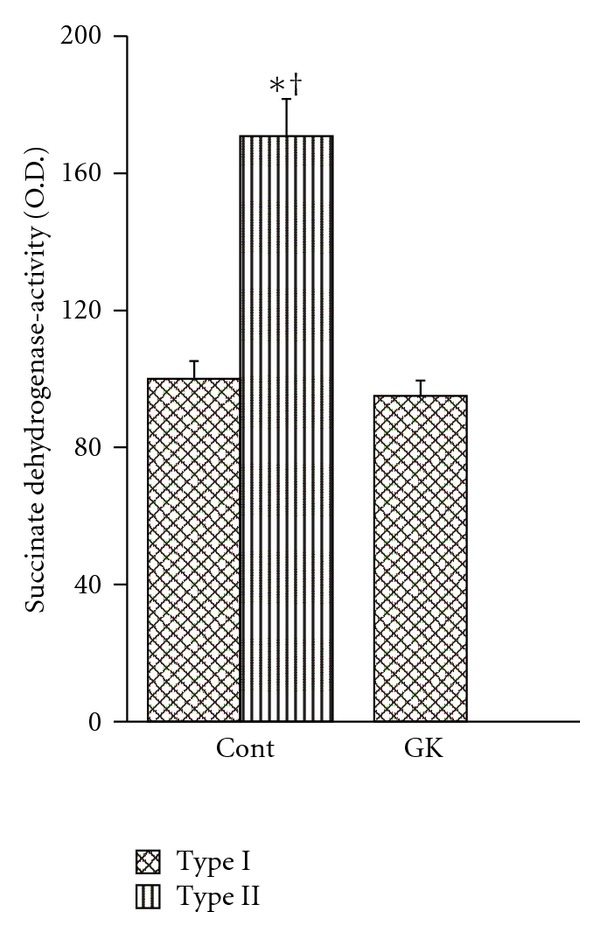
The mean SDH activities in the type I and type II fibers of the Cont and GK rats. The SDH activity of type II fibers of the Cont rats is significantly higher than that of type I fibers of the Cont and GK rats. *P < 0.05 compared with type I fibers of Cont rats. ^†^ *P* < 0.05 compared with type I fibers of GK rats.

 Staining for SDH activity showed that type II fibers of the soleus muscle have higher activity than type I fibers. This resulted in a different composition of fiber type for the GK and Cont rats because the soleus muscle of the GK rats have only type I fibers (Figures 2(b) and 2(e)). The SDH activity in the type II fibers of the soleus muscle in the Cont rats was 170.8 ± 10.9 (O.D.), while the SDH activity was 95.0 ± 4.5 and 100 ± 5.2 in the type I fibers of the GK and Cont rats, respectively. There was no significant difference in the SDH activity in the type I fibers of the soleus muscle for the GK and Cont rats (Figure 3). Accordingly, the net activity of oxidative enzymes was significantly lower in the muscles of the GK rats than in that of the Cont rats. 

### 3.4. Capillary Architecture, Density, and Volume

 The mean capillary density of the soleus muscle was 649.0 ± 59.8/mm^2^ in the GK rats and 657.0 ± 53.6/mm^2^ in the Cont rats (*P* < 0.05) (Table 1; Figures 2(c) and 2(f)). 

**Figure 4:**
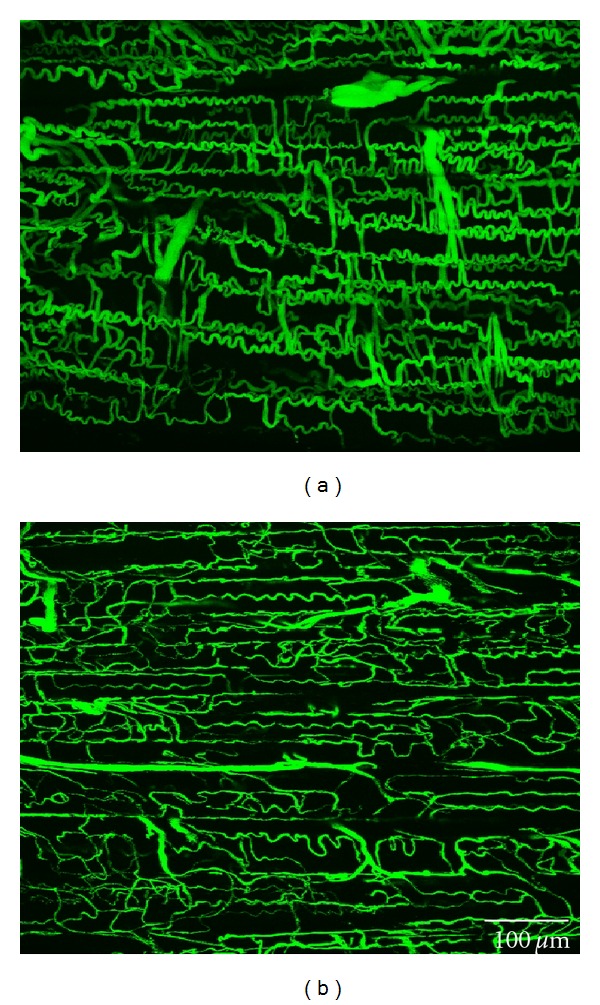
Confocal laser scanning microscopy images of the capillaries and anastomoses in the soleus muscle of the Cont (a) and GK (b) rats. Scale bar: 100 *μ*m.

**Figure 5:**
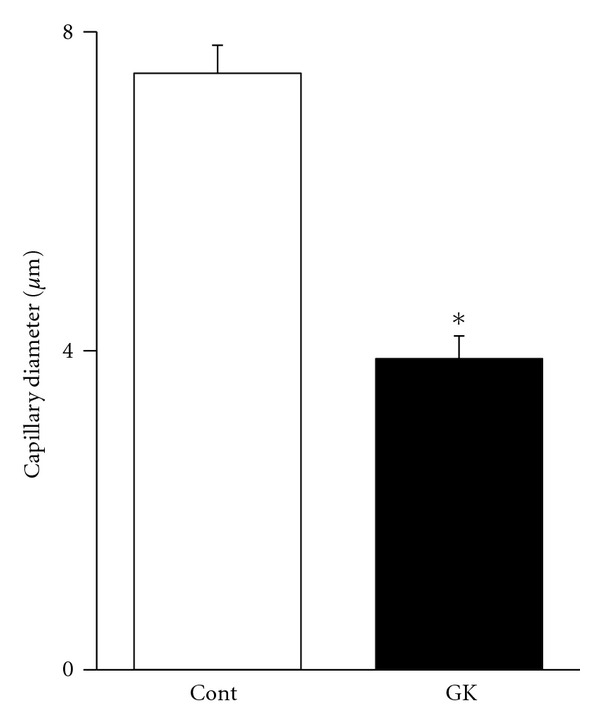
The mean capillary diameter in the soleus muscle of the Cont and GK rats. The capillary diameter in the soleus muscle of the Cont rats was thicker than that in the GK rats.

**Figure 6:**
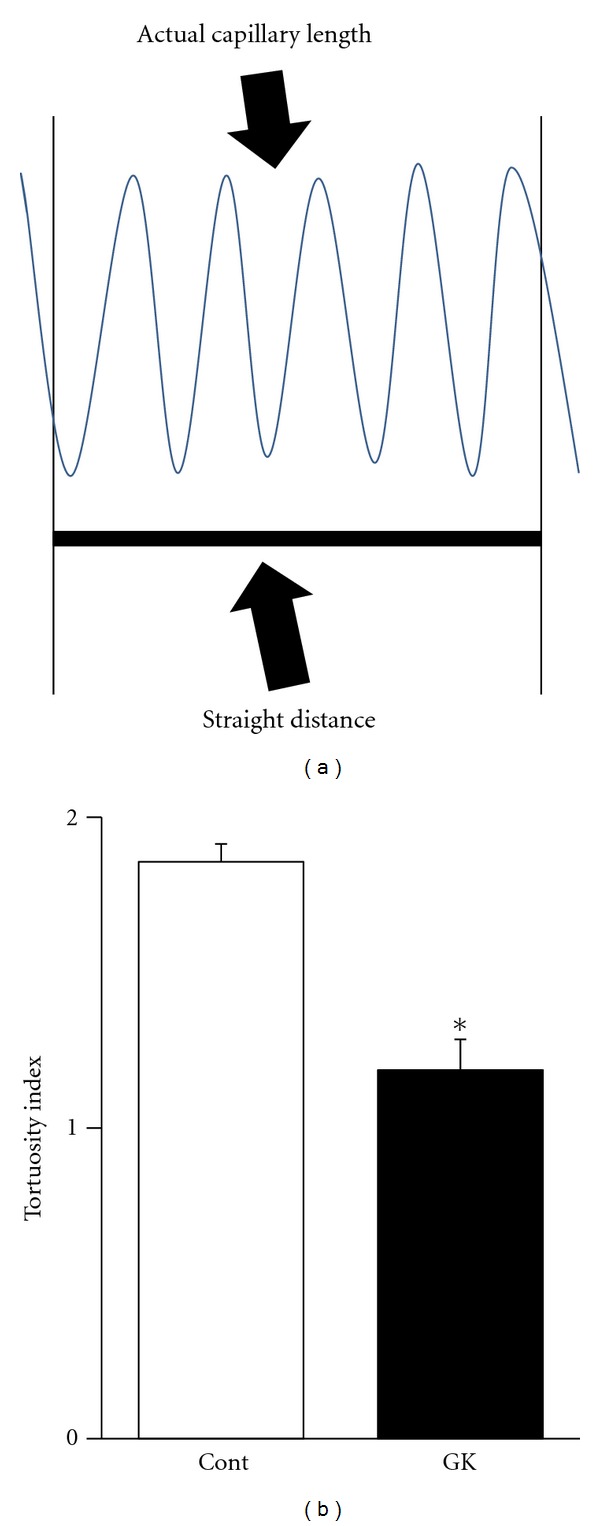
Tortuosity index was calculated by dividing the actual capillary length by the straight distance (a). The mean tortuosity index of the Cont rats was greater than that in the GK rats (b). *P < 0.05.

The 3D reconstructed CLSM images showed the capillary architecture in the soleus muscles of the GK and Cont rats (Figure 4). In the muscles of both types of animals, longitudinal capillaries ran along the muscle fibers, and adjacent longitudinal capillaries were connected by several interconnecting anastomoses. The longitudinal capillaries running parallel to muscle fibers showed a tortuous course in both rats. However, the course in the muscles of the GK rats seems less tortuous and thinner than that in the muscles of the Cont rats, whose longitudinal capillaries waved with small amplitude. The capillary diameter in the soleus of the GK rats was 3.90 ± 0.28 *μ*m and that in the Cont rats was 7.48 ± 0.35 *μ*m (Figure 5). Tortuosity index was calculated by dividing the actual capillary length by the straight distance (Figure 6(a)). The tortuosity index of the soleus muscle was smaller in the GK rats than in the Cont rats (1.19 ± 0.10 versus 1.86 ± 0.06, *P* < 0.05) (Figure 6(b)).

**Figure 7:**
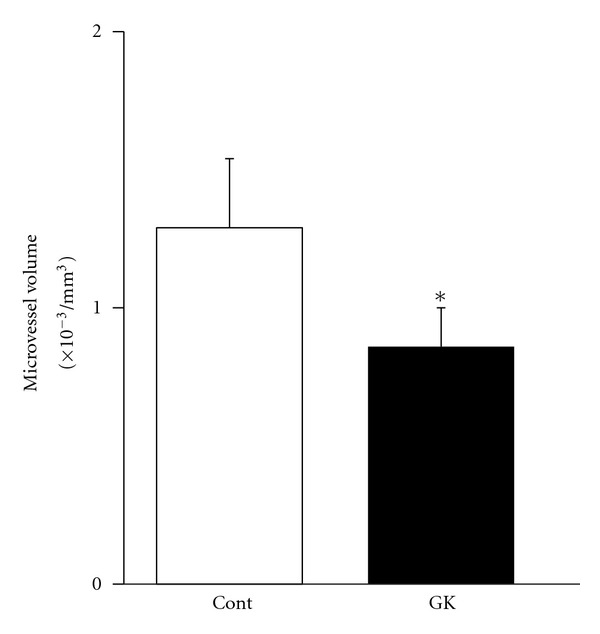
Data from the morphological measurement of the CLSM images by the NIH Image software. The mean microvessel volume of the GK rats was significantly lower than that in the Cont rats. *P < 0.05.

 The mean microvessel volume in the soleus muscle (/mm^3^) was lower in the GK rats than in the Cont rats (1.29 ± 0.25 × 10^−3^/mm^3^ versus 0.86 ± 0.14 × 10^−3^/mm^3^, *P* < 0.05) (Figure 7).

## 4. Discussion

Type 2 diabetes is associated with impaired glucose metabolism and insulin insensitivity. The percentage of high-oxidative fibers is one of the important factors related to whole-body insulin sensitivity [13, 23] Several studies have shown impaired metabolic properties specific to the fiber type in the skeletal muscles of patients and animal models with type 2 diabetes, in particular with regard to high-oxidative fibers. In addition, fiber-type transformation has been observed in the soleus muscle of obese Zucker diabetic fatty rats [8]. The capillary content of skeletal muscle is an important determinant of the efficiency of the exchange of oxygen between the red blood cells and muscle fibers. High-oxidative muscles, in general, are more heavily recruited and have a higher capillary content than the low-oxidative muscles [24, 25]. Our previous study demonstrated that the capillary volume is higher in the high-oxidative soleus muscle than in the low-oxidative extensor digitorum longus muscle [17]. In addition, we have shown regressed capillaries in diabetic muscles [20]. Thus, decreased high-oxidative muscle fibers may play a role in reducing capillary content in the nonobese type 2 diabetic rats. In the present study, decreased percentage of the high-oxidative fibers in the soleus muscle of nonobese type 2 diabetic rats was linked to the fiber-type transformation and regression of the muscle capillary network.

The soleus muscle of the rat is composed of type I and type II muscle fibers, depending on the MyHC isoform [7, 11, 26]. The soleus muscles of the 36-week-old Cont rats were composed of a high percentage of type I fibers and a low percentage of type II fibers. However, there were no type II fibers in the soleus muscles of the age-matched GK rats. Previous studies have shown that Otsuka Long-Evans Tokushima Fatty (OLETF) rats at 21 weeks [10] and GK rats at 20 weeks [11] have a different pattern of muscle fiber type in the soleus muscles compared with rats without diabetes mellitus. 

The present study also demonstrated that the SDH activity of type II fibers in the soleus muscle of the Cont rats was significantly higher than that of type I fibers of the Cont rats and GK rats. The SDH activity level shows the degree of the activity of mitochondria, that is, oxygen demand [9–11, 27]. Several studies have demonstrated that the SDH activity of type II fibers is higher than that of type I fibers in the soleus muscle [8, 12]. In the present study, the overall oxidative enzyme activity in the muscle was significantly lower in the GK rats than in the Cont rats because the soleus muscle of the GK rat lacks high-oxidative type II fibers. Consequently, the activity of mitochondria decreases, and the oxygen demand decreases.

Two-dimensional capillary density is not significantly different between the GK and Cont rats. However, 3D capillary diameter, capillary tortuosity, and microvessel volume in the GK rats were significantly lower than in the Cont rats. The differences in the capillary architecture of the GK and Cont rats may have arisen in order to meet the different levels of oxygen demand; since the muscles of the GK rats have lower oxygen demand, the capillary requirement is diminished [2, 17, 18, 21].

In conclusion, the soleus muscle of GK rats has altered muscle fiber distribution, decreased SDH activity, and reduced capillary content compared to that of the Cont rats. The distribution of fiber type in the soleus muscle of the GK rat was type I alone, with no high-oxidative type II fibers present. Therefore, the entire oxygen demand of the whole muscle fiber is decreased in the soleus muscle of the GK rats. Consequently, the capillary network in the nonobese type 2 diabetic rats regressed. These results indicate that a decrease in the high-oxidative muscle fibers plays a role in reducing the capillary content in the nonobese type 2 diabetic rats.

## Figures and Tables

**Table 1:** Body and muscle parameter data in rats soleus.

	Body mass (g)	Muscle mass of soleus (mg)	FCSA (*μ*m^2^)	CD (number/mm^2^)
Cont	381.4 ± 3.3	134.0 ± 4.1	2502.5 ± 165.0	657.0 ± 53.6
GK	415.4 ± 10.8^#^	163.6 ± 6.6^#^	2580.9 ± 160.1	649.0 ± 59.8

Values are means ± S.E.M. *n* = 8. FCSA: fiber cross-sectional area; CD: capillary density; ^#^Significantly different from con (*P* < 0.05).
